# Mechanism of wavelength conversion in polystyrene doped with benzoxanthene: emergence of a complex

**DOI:** 10.1038/srep02502

**Published:** 2013-08-26

**Authors:** Hidehito Nakamura, Yoshiyuki Shirakawa, Hisashi Kitamura, Nobuhiro Sato, Osamu Shinji, Katashi Saito, Sentaro Takahashi

**Affiliations:** 1 Kyoto University, 2 Asashiro-Nishi, Kumatori-cho, Sennan-gun, Osaka 590-0494, Japan; 2 National Institute of Radiological Sciences, 4-9-1, Anagawa, Inage-ku, Chiba 263–8555, Japan; 3 Kuraray Co., Ltd., 2–28, Kurashiki-Cho Tainai, Niigata, 959–2691, Japan

## Abstract

Fluorescent guest molecules doped in polymers have been used to convert ultraviolet light into visible light for applications ranging from optical fibres to filters for the cultivation of plants. The wavelength conversion process involves the absorption of light at short wavelengths followed by fluorescence emission at a longer wavelength. However, a precise understanding of the light conversion remains unclear. Here we show light responses for a purified polystyrene base substrates doped with fluorescent benzoxanthene in concentrations varied over four orders of magnitude. The shape of the excitation spectrum for fluorescence emission changes significantly with the concentration of the benzoxanthene, indicating formation of a base substrate/fluorescent molecule complex. Furthermore, the wavelength conversion light yield increases in three stages depending on the nature of the complex. These findings identify a mechanism that will have many applications in wavelength conversion materials.

Due to considerable losses by absorption and reflections, short-wavelength light transmission over long distances in optical fibres is challenging^1^,^2^. Moreover, plants do not efficiently utilize short-wavelength sunlight during photosynthesis. To overcome these problems, wavelength conversion materials have been developed. These materials are polymers doped with fluorescent guest molecules that absorb light at shorter wavelengths and re-emit it at longer wavelengths^3^,^4^. However, there are aspects of this process that are not fully understood.

Advanced refining techniques enable the production of high purity substrates and the control of fluorescent molecule concentrations in wavelength conversion materials^5^. Here we characterize in detail the light response for a purified wavelength conversion material by varying the concentration of a fluorescent benzoxanthene derivative in a polystyrene base substrate over four orders of magnitude. Based on the fluorescent spectra and light yields over the large benzoxanthene concentration range, we reveal a complex between the polystyrene and the fluorescent benzoxanthene. The repeat unit for polystyrene and the structure of the benzoxanthene derivative are shown in Fig. 1 and 2, respectively.

## Results

**Figure 3:**
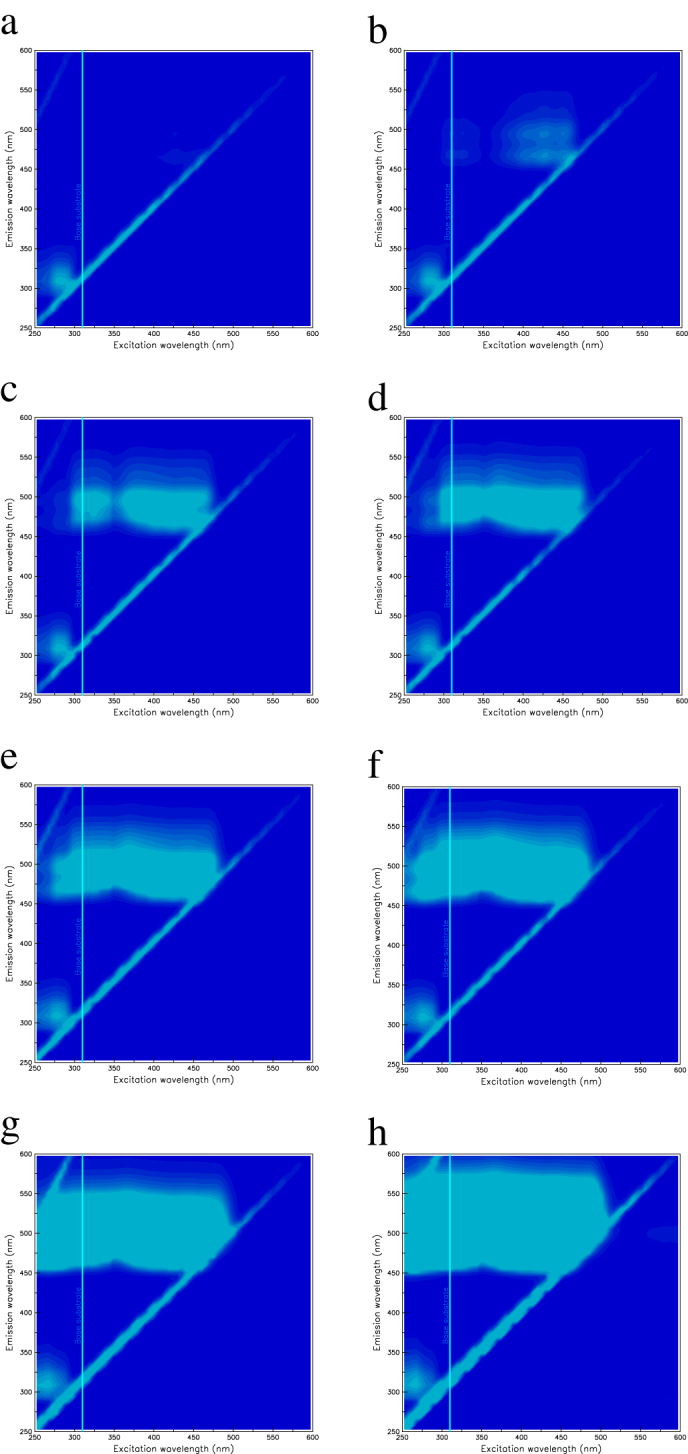
Two-dimensional fluorescence spectra of polystyrene doped with benzoxanthene. The concentrations of the benzoxanthene derivatives are (a) 0.0002 wt%, (b) 0.002 wt%, (c) 0.02 wt%, (d)0.05 wt%, (e) 0.1 wt%, (f) 0.2 wt%, (g) 0.5 wt% and (h)1.0 wt%. The 310-nm maximum (peak) in the emission wavelength for the pure, undoped base substrate is denoted by the light blue line in each x-axis.

**Figure 4:**
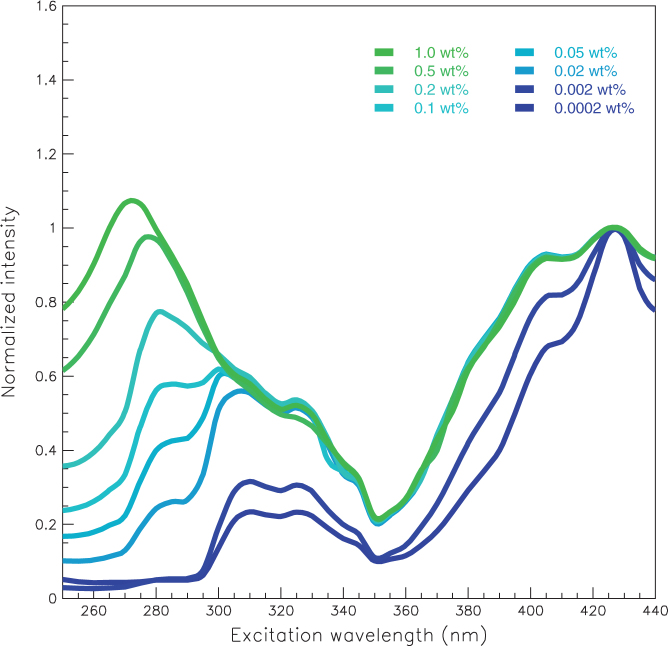
Excitation spectra for polystyrene doped with various concentrations of benzoxanthene, as monitored at 465 nm. The excitation peaks are normalized at 425 nm.

Two-dimensional fluorescence spectra for polystyrene samples with various concentrations of benzoxanthene are shown in Fig. 3. Two distributions are observed: one is in the 310-nm region due to the polystyrene, and the other is around 465 nm due to the benzoxanthene. Fig. 4 shows the excitation spectrum monitored at 465 nm for each sample. The spectra clearly do not overlap, and significantly differ between the 0.02 to 0.2 wt% concentrations of benzoxanthene. These results indicate a possible interaction between the polystyrene and the benzoxanthene. Furthermore, excitation peaks below 350 nm emerge as the benzoxanthene concentration increases.

**Figure 5:**
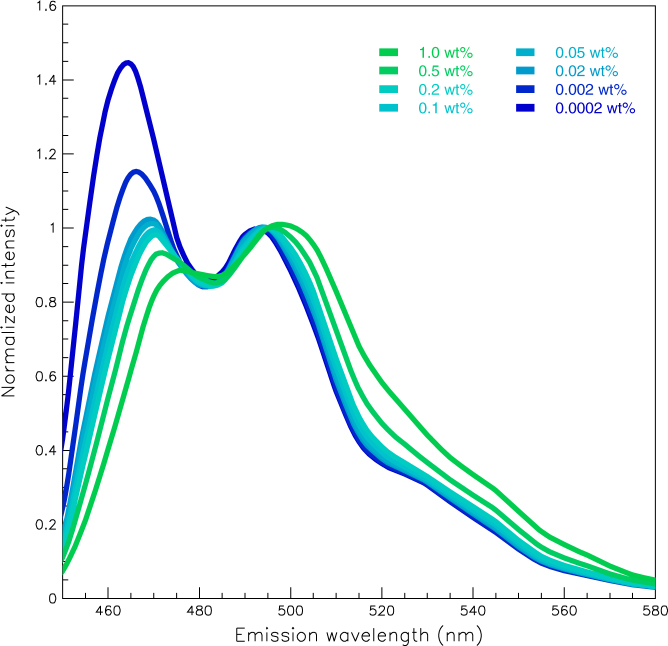
Emission spectra due to 425-nm excitation for polystyrene doped with various concentrations of benzoxanthene. The emission peaks are normalized at 495 nm.

**Figure 6:**
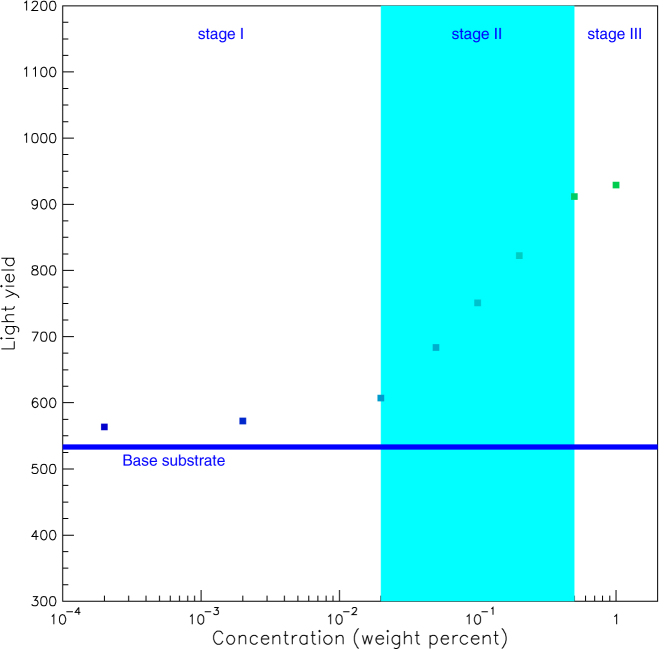
Light yield for polystyrene doped with benzoxanthene. The light yield for the undoped polystyrene base substrate is denoted by the blue line. Each error bar is within each data point.

Fig. 5 shows the emission spectrum due to 425-nm excitation for each sample. We see that UV light is converted to blue-green light by adjusting the fluorescent concentrations. Fig. 6 shows the light yield for each wavelength conversion sample, where the yield for undoped polystyrene base substrate is given by the blue line. The light yield increases in three distinct stages with the benzoxanthene concentration, in an S-shaped curve. The three stages in the light yields can also be seen in Fig. 3. This observation, as with the excitation spectra, indicates that there is an interaction between the polystyrene and the benzoxanthene. The light blue line in each x-axis denotes the 310-nm maximum in the undoped polystyrene emission. The line for the spread of the distribution is the boundary between the first and second stages. When the distribution spreads to 250 nm, there is a shift from the second to the third stage.

These results indicate that the polystyrene and benzoxanthene are not independently excited and de-excited. Instead, complexes develop over three stages depending on the benzoxanthene concentration. At the first stage, the interaction between the polystyrene and the benzoxanthene is weak and each behaves independently. The second stage indicates a definite interaction and a loss of independence. In the third stage, the interaction is strong, the independence is completely lost, and there is a possibility of concentration quenching^6^.

## Discussion

The conversion mechanism revealed at wide fluorescent molecule concentrations expands the scope of applications^7^,^8^,^9^,^10^,^11^,^12^,^13^,^14^. For example, optical fibres that are used for wavelength conversion have 0.01–0.03 wt% fluorescent doping^15^. The excitation distribution of the fluorescence now spreads to short wavelength regions (*e.g.,* 250 nm) at those concentrations. When using such fibres, we should therefore take care of both the targeted light (370–480 nm) and short-wavelength light external to the fibres, or the light emitted from the base substrate. On the other hand, the wavelength conversion materials have a great potential to convert internal or external ultraviolet light to visible light by adjusting fluorescent guest molecule concentrations.

In summary, we have characterized polystyrene base substrates doped with the fluorescent benzoxanthene over a wide concentration range. Complexes between the polystyrene and the benzoxanthene emerged, indicating that wavelength conversions have complicated mechanisms.

These findings have a great potential to improve the overall performance of the wavelength conversion materials. It is also important to investigate the wavelength conversion materials formed by other base substrates/fluorescent molecules^16^,^17^,^18^.

## Methods

Styrene monomers were refined *via* reduced-pressure distillation and mixed with different concentrations of benzoxanthene (0, 0.0002, 0.002, 0.02, 0.05, 0.1, 0.2, 0.5, 1.0 wt%). The purity of the benzoxanthene was more than 99.1%, and the purity of the polystyrene was more than 99.9%. These solutions were injected through 0.2-μm pore filters into glass reaction vessels and then sealed under vacuum. The solutions were thermally polymerized in an oil bath held first at 100°C and then elevated to 150°C. Colouration brought about by heat deterioration or oxygen contamination was minimized. The average molecular weight of the polystyrene doped with the benzoxanthene was approximately 300,000 at a 99.5% polymerization rate. Each wavelength conversion material was cut into 62 × 62 × 10 mm samples and then polished.

Fluorescence spectra were acquired with a spectrophotometer (F-2700; Hitachi High-Technologies Co., Ltd.). To avoid a bias to the light yield caused by light absorption and emission by the polystyrene and the benzoxanthene, the yields were determined with a ^207^Bi radioactive source (BIRB4391; High Technology Source Ltd.) and a photomultiplier tube (PMT, R878-SBA; Hamamatsu Photonics Co., Ltd.). Monoenergetic internal conversion electrons from the radioactive source generated light emission in the samples. The PMT had the equivalent quantum efficiency as the two fluorescence regions of the polystyrene and the benzoxanthene. The 62 × 62 mm face of each sample was in contact with the PMT photocathode *via* optical grease (EJ-550; Eljen Technology), and the radioactive source was placed in the centre of the opposite face.

## Author Contributions

H.N. planned the research; H.N., Y.S., H.K., N.S. and S.T. performed research; O.S. and K.S. prepared the sample. All authors discussed the results and commented on the manuscript.

## Figures and Tables

**Figure 1:**
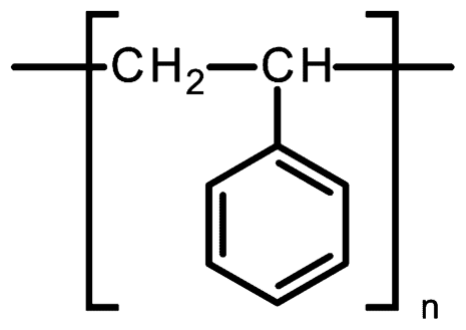
Repeat unit for polystyrene.

**Figure 2:**
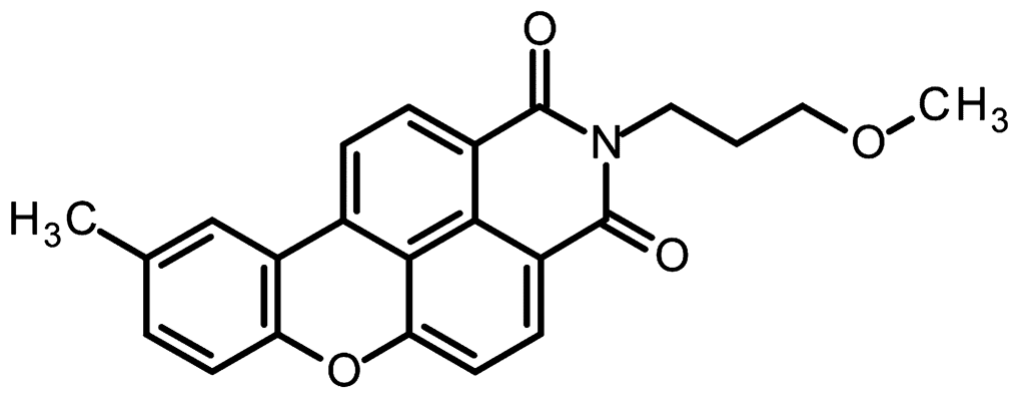
Structure of the benzoxanthene derivative.
